# Subcellular Antigen Location Influences T-Cell Activation during Acute Infection with *Toxoplasma gondii*

**DOI:** 10.1371/journal.pone.0022936

**Published:** 2011-07-28

**Authors:** Beth Gregg, Florence Dzierszinski, Elia Tait, Kimberly A. Jordan, Christopher A. Hunter, David S. Roos

**Affiliations:** 1 Department of Biology, University of Pennsylvania, Philadelphia, Pennsylvania, United States of America; 2 Department of Pathobiology, University of Pennsylvania, Philadelphia, Pennsylvania, United States of America; University of Louisville, United States of America

## Abstract

Effective control of the intracellular protozoan parasite *Toxoplasma gondii* depends on the activation of antigen-specific CD8^+^ T-cells that manage acute disease and prevent recrudescence during chronic infection. T-cell activation in turn, requires presentation of parasite antigens by MHC-I molecules on the surface of antigen presenting cells. CD8^+^ T-cell epitopes have been defined for several *T. gondii* proteins, but it is unclear how these antigens enter into the presentation pathway. We have exploited the well-characterized model antigen ovalbumin (OVA) to investigate the ability of parasite proteins to enter the MHC-I presentation pathway, by engineering recombinant expression in various organelles. CD8^+^ T-cell activation was assayed using ‘B3Z’ reporter cells *in vitro*, or adoptively-transferred OVA-specific ‘OT-I’ CD8^+^ T-cells *in vivo*. As expected, OVA secreted into the parasitophorous vacuole strongly stimulated antigen-presenting cells. Lower levels of activation were observed using glycophosphatidyl inositol (GPI) anchored OVA associated with (or shed from) the parasite surface. Little CD8^+^ T-cell activation was detected using parasites expressing intracellular OVA in the cytosol, mitochondrion, or inner membrane complex (IMC). These results indicate that effective presentation of parasite proteins to CD8^+^ T-cells is a consequence of active protein secretion by *T. gondii* and escape from the parasitophorous vacuole, rather than degradation of phagocytosed parasites or parasite products.

## Introduction

Class I Major Histocompatability Complex (MHC-I) molecules present peptides generated by proteasomal degradation in the cytosol and transport into the endoplasmic reticulum, or by cross-presentation of endo/phagocytosed material [1], [2]. During infection, MHC-I antigen presentation is responsible for the activation and expansion of specific CD8^+^ T-cells, and is involved in the immune response to diverse intracellular pathogens, including viruses, bacteria, and microbial eukaryotes [3], [4]. CD8^+^ T-cells are required for the control of the protozoan parasite *Toxoplasma gondii* during its acutely lytic ‘tachyzoite’ stage, and (directly or indirectly) during the chronic ‘bradyzoite’ stage characterized by latent cysts in the muscle, brain and other tissues [5], [6]. Activated T-cells recognize and destroy both *T. gondii* parasites and parasite-infected cells, and also produce IFN-γ, activating reactive oxygen pathways [7]–[9].

The route by which *T. gondii* antigens reach the endoplasmic reticulum for loading onto MHC-I is not fully understood, as these parasites reside within a specialized intracellular ‘parasitophorous vacuole’ (PV) distinct from the phagocytic/endocytic pathway and the host cell cytoplasm. Presentation is dependent on host cell immunoproteasomes, TAP, and ERAAP [10]–[12], indicating that parasite antigens must reach the host cell cytosol, and several pathways have been proposed, including cross-presentation of phagocytosed parasite material, degradation of the PV membrane, secretion of parasite proteins outside of the PV, and fusion of the PV with the host cell ER [13]–[15]. Various immunogenic *T. gondii* antigens are known, including proteins secreted from the dense granules and rhoptries, but responses are often both parasite and host strain-specific [9], [12], [16], [17]. In order to address the route of *T. gondii* antigen entry into the MHC-I presentation pathway, we have examined CD8^+^ T-cell activation following infection with parasites engineered to target the well-characterized antigen ovalbumin to various locations, including the parasite cytoplasm, mitochondrion, inner membrane complex, plasma membrane, and the parasitophorous vacuolar space.

## Results

### Generation of transgenic parasite expressing organelle specific OVA antigen

**Figure 1:**
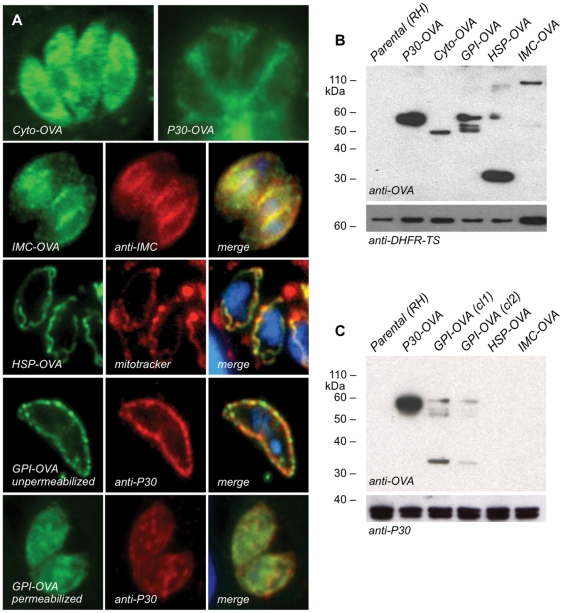
Stable *T. gondii* parasite lines expressing transgenic OVA antigen. (A) OVA protein fused to endogenous targeting signals correctly traffics OVA antigen (green) to specific organelles in *T. gondii* tachyzoites, including: the cytoplasm (row 1, left), the parasitophorous vacuole (row 1, right), the inner membrane complex (row 2), the mitochondrion (row 3), and the parasite membrane (row 4). Permeabilization reveals intracellular, as well as membrane-bound, GPI-OVA (row 5). Co-localization markers (red) include: anti-IMC1 antibody, mitotracker, and anti-P30 antibody, as labeled. Protein expression in stable transgenic parasites (B) and culture supernatants (C) was analyzed by immunblotting, using antibodies directed against OVA and P30 (loading control).

To explore whether antigen access to the MHC-I presentation pathway is affected by subcellular location of antigen within *T. gondii*, RH strain *T. gondii* was engineered to stably express the model antigen OVA (amino acids 140–386), fused to various organelle-targeting sequences as described under Methods. The rationale behind these experiments was to help distinguish between cross-presentation of phagocytosed antigen, *versus* translocation of antigen across the parasitophorous vacuole where intracellular *T. gondii* parasites reside (a compartment distinct from the endophagocytic system; [18]). As shown in Fig. 1A (top row), expression of ovalbumin without additional targeting signals results in cytoplasmic localization (Cyto-OVA), while fusion to a signal sequence results in secretion into the parasitophorous vacuole (P30-OVA), as previously described by Pepper *et al* [19]. Co-localization of additional OVA fusion proteins with well-characterized markers showed proper targeting to the inner membrane complex (IMC-OVA, row 2), the mitochondrion (HSP-OVA, row 3), or the cell surface, using a GPI anchor (GPI-OVA, row 4). Antibodies to OVA label the surface of non-permeabilized, extracellular GPI-OVA parasites, indicating targeting to the parasite membrane (row 4); permeabilization prior to staining also reveals OVA associated with internal secretory organelles (ER, Golgi, vesicles), presumably *en route* to the plasma membrane (row 5).

As antigen load is known to be important during infection [20], OVA levels were assessed by immunoblotting of parasites (Fig. 1B) and infected culture supernatants (Fig. 1C). Levels of OVA produced by individual parasite strains ranged from 4.5 to 21 ng/10^6^ parasites. Secreted antigen was detected only in P30-OVA and GPI-OVA culture supernatants (Fig. 1C). We presume that significant quantities of GPI-OVA protein are shed from the parasite surface during parasite gliding motility, as has been reported for the endogenous P30 protein [21]. No secreted antigen was detected in culture supernatants following HSP-OVA or IMC-OVA infection. Cyto-OVA was not included on this Western blot, but no secreted OVA was detected in Pru Cyto-OVA transgenics (not shown), and note that no secreted antigen is visible in Fig. 1A (top left). *In vitro* assays indicate similar rates of replication for all of the transgenics described in this report, and preliminary real-time PCR analysis of liver and spleen samples from infected mice indicates <1.5-fold difference in parasite burden (data not shown).

### Antigen presentation and T-cell activation *in vitro* correlate with antigen secretion

**Figure 2:**
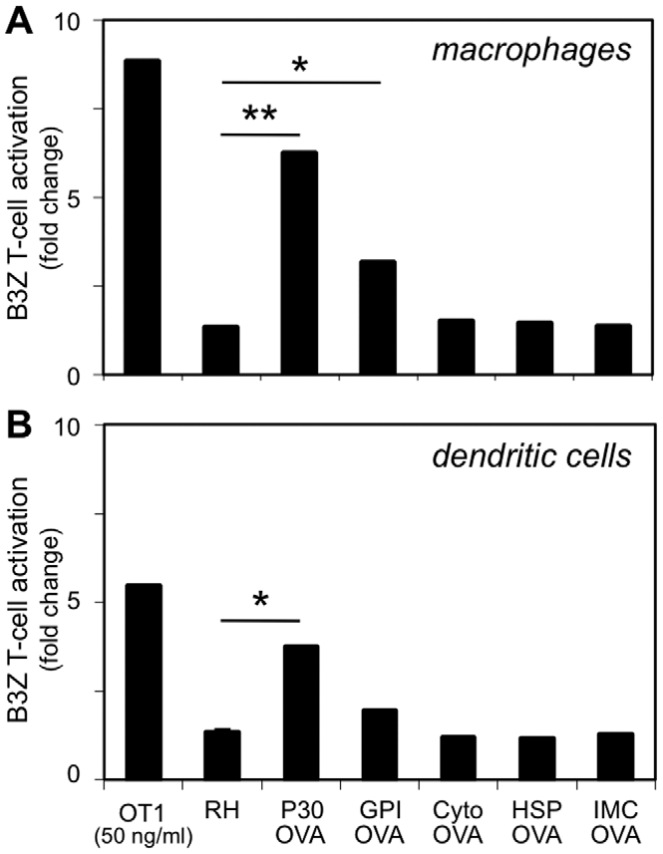
Activation of OVA-specific B3Z T-cells by *T. gondii* expressing OVA antigen. Bone marrow derived macrophages (A) and dendritic cells (B) were stimulated with OT-I peptide or infected with *T. gondii* expressing OVA in various subcellular compartments (Fig. 1), and co-cultivated with B3Z T-cells in medium containing the ß-galactosidase substrate CPRG. Absorbance at 565 nm is represented relative to controls treated with media alone (average of three replicate assays). Results shown are representative of three independent experiments. Asterisks indicate *p*<0.05 (*) or *p*<0.005 (**), based on the students T-test statistic.

The ability of OVA transgenic lines to induce MHC-I antigen presentation *in vitro* was determined using the ‘B3Z’ CD8^+^ T-cell hybridoma line [22]. Recognition of the OVA antigenic epitope ‘SIINFEKL’ in the context of the H-2K^b^ restricted MHC-I mouse background causes activation of these cells to produce ß-galactosidase, which is readily detected by conversion of CPRG to a purple reaction product. Activated (IFN-γ treated) bone marrow-derived macrophages were able to present antigen (activate B3Z cells) when infected with *T. gondii* parasites secreting OVA (P30-OVA and GPI-OVA transgenics), but not parasites harboring intracellular OVA antigen (Cyto-OVA, HSP-OVA, IMC-OVA), as shown in Fig. 2A. Significant activation was also seen in bone marrow-derived dendritic cells following infection with P30-OVA transgenics (Fig. 2B); an increase was observed in GPI-OVA-infected dendritic cells as well, although this failed to achieve statistical significance at *P*<0.05. The lower levels of activity observed using GPI-OVA transgenics likely reflect the lower levels of OVA secreted by this parasite line (Fig. 1C). T-cell activation was strictly dependent on the presence of live *T. gondii* parasites: heat-killed parasites were readily taken up by activated macrophages, but these effectors failed to activate B3Z cells (data not shown).

### Activation of naïve OT-I cells *in vivo* correlates with OVA secretion

**Figure 3:**
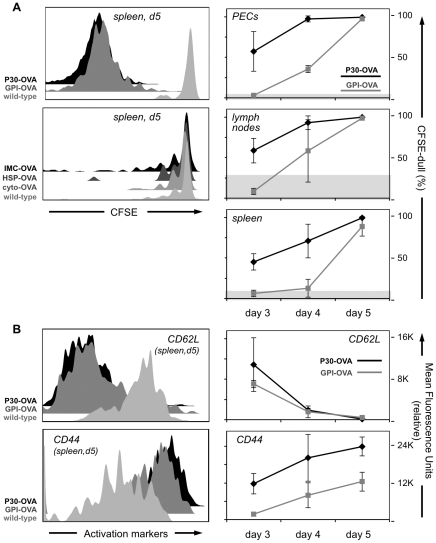
Proliferation of adoptively transferred OT-I T-cells after infection with OVA-expressing *T. gondii*. (A) CFSE levels of Thy1.2+ OT-I T-cells after murine infection with *T. gondii* expressing secreted or GPI-anchored OVA (top left) or intracellular OVA antigens (bottom left). Right-hand panels indicate the time course of OT-I activation in various organs (% CFSE-dull cells) following infection with P30-OVA or GPI-OVA. (B) CD62L (top) and CD44 (bottom) levels on Thy1.2+ OT-I T-cells after mice were infected with *T. gondii* expressing either secreted or GPI-anchored OVA (left, histograms; right, time course).

In order to determine whether the activation of B3Z T-cells by *T. gondii*-expressed OVA antigen *in vitro* reflects T-cell activation *in vivo*, non-activated OVA specific CD8^+^ T-cells (OT-I cells) were labeled with carboxy-fluoresceine succinimidyl ester (CFSE) and transferred into naïve mice. Five days after infection with *T. gondii* transgenics expressing OVA or wild-type controls, cells from the peritoneum, parathymic lymph node, and spleen were assayed for CFSE dilution and T-cell activation markers, as shown in Figure 3.

OT-I cells from uninfected animals, or mice infected with wild-type RH strain *T. gondii*, showed no diminution of CFSE levels, indicating that the T-cells were not activated by parasite infection *per se* over the course of this experiment (Fig. 3A, light shading). All mice infected with either of the parasite lines secreting OVA (P30-OVA, GPI-OVA) displayed marked dilution of CFSE (Fig. 3A upper left, intermediate and dark shading). The kinetics of CFSE dilution in P30-OVA and GPI-OVA differed, however (Fig. 3A, right-hand panels): P30-OVA parasites activated T-cell proliferation as early as 3 days post-infection in all three compartments (peritoneal exudate (PECs), lymph node, spleen), and by day 4 essentially all OT-I cells displayed reduced CFSE levels. In contrast, T-cell activation by GPI-OVA parasites was first observed at day 4 and while all OT-I cells had divided by day 5, CFSE dilution still lagged slightly behind that observed following P30-OVA parasite infection (upper left). This delay likely reflects slower accumulation of antigen due the slow release of GPI-anchored OVA from the parasite cell surface. Further indication of OT-I cell activation during infection is provided by the down-regulation of CD62L and up-regulation of CD44 (Fig. 3B) and CD69 (not shown). Time-course assays indicate similar kinetics of OT-I cell activation, whether measured by CFSE dilution or activation markers (compare the right-hand panels of Fig. 3A with Fig. 3B). Note that while mean fluorescence levels shows a significant lag in CD44 up-regulation by GPI-OVA parasites (Fig. 3B, bottom right), the histogram (bottom left) indicates that the spectrum of CD44 expression in the GPI-OVA sample is closer to the P30-OVA sample than the wild-type control.

In contrast to the B3Z assay (Fig. 2), which showed no detectable activation of T-cells by intracellular OVA, the more sensitive *in vivo* system shows low levels of OT-I cell activation by these parasites (Fig. 3A, lower left). At 5 days post infection, 50% of OT-I cells showed a history of proliferation in the IMC-OVA sample, based on reduced CFSE levels (note, however that this reflects proliferation of <50% of the starting population, as cell numbers double with each division). These proliferating cells also displayed up-regulation of CD44 and CD69, and lower levels of surface CD62L (not shown). 30% of OT-I cells in the HSP-OVA sample showed evidence of proliferation, while 15% showed evidence of proliferation in response to Cyto-OVA; proliferation was observed in only 9% of OT-I cells in response to wild-type parasite infection. These results confirm that *T. gondii* antigens secreted into the parasitophorous vacuole are readily presented on MHC-I. Intracellular antigens are far less prone to MHC-I presentation (and may derive from phagocytosis and cross-presentation of dead parasites and parasite debris; see below for further discussion).

## Discussion

Activation of CD8^+^ T-cells is known to be critical for the control of *T. gondii* infection [5], [23]. In order to further clarify the basis of MHC-I antigen presentation during infection, OVA was expressed in various subcellular compartments (Fig. 1), including three locales within the parasite (the cytoplasm, inner membrane complex, and mitochondrion) and two external locales (anchored to the plasma membrane via a GPI anchor, and secreted into the parasitophorous vacuole). Efforts to stably express OVA antigen in the rhoptries and micronemes were unsuccessful (see Methods); while we (and others) have successfully targeted various reporters into these organelles in transient transfectants, stable transgenics are often more difficult to obtain, and these proteins typically fail to secrete [24]. Our results demonstrate that subcellular localization of antigen matters: surface and secreted antigen is readily presented, while internal antigen is not (Figs. 2 & 3). While surface antigens may be released at low levels from parasites prior to invasion by either dense granule secretion or protein shedding [21], [25], [26], the failure to present OVA from internal compartments, and the requirement for living parasites, indicates that biologically relevant antigen loading occurs only during active infection, rather than by phagocytosis of parasites or parasite debris from lysed host cells. These results support previous observations with parasites secreting OVA or β-galactosidase [11], [27], [28], although evidence for cross-presentation by uninfected dendritic cells has also been reported during Prugniaud P30-OVA infection [13].

Activation of OT-I cells induced by presentation of OVA antigen secreted into the parasitophorous vacuole occurs as early as day 3 post-infection. These data are consistent with previous reports that CD8 responses against pathogens (including *T. gondii*) are initiated within 3 days of infection [13], [29]. By 5 days post-infection, antigen-specific OT-I cells had proliferated extensively in response to secreted OVA, while responses to intracellular OVA were just getting underway (Fig. 3).

Although OVA is not a native parasite antigen, previous studies have highlighted the importance of secreted *T. gondii* proteins in CD8^+^ T-cell responses to infection with various parasite strains (P30/SAG1 [8], [9], [30], GRA6 [12], GRA4 & ROP7 [16], TGD057 [17]). In addition to secreted antigens, the observed CD8^+^ T-cell response to GPI-OVA, while slightly lower than responses to P30-OVA (Fig. 3), suggests the possible importance of the large SAG/SRS family [31] as subdominant epitopes in *Toxoplasma* infection. These findings are also consistent with observations on other intracellular pathogens: antigens secreted by either bacteria (*Listeria monocytogenes*) or protozoa (*Leishmania major*, *Trypanosoma cruzi*) are more effective in activating CD8^+^ T-cells than intracellular antigen [32]–[34]. In the *Listeria* system, it has also been shown that T-cells stimulated by intracellular antigen are limited in their ability to lyse infected cells, presumably because intracellular antigens are unlikely to be presented by infected target cells [32].

Both *Listeria* and *T. cruzi* live within the host cell cytoplasm, but *T. gondii* is found within the ‘parasitophorous vacuole’, a specialized intracellular compartment distinct from the endo-phagocytic pathway [18]. The fact that secreted OVA is presented by infected cells therefore implies that this antigen must escape from the parasitophorous vacuole into the cytoplasm. The mechanism by which OVA reaches the host cell cytoplasm is uncertain, but there is ample precedent for traffic out of the parasitophorous vacuole [11], [35]–[37]. Previous studies have shown that presentation of secreted OVA is dependent on TAP [11], [27], which mediates transport from the cytoplasm into the endoplasmic reticulum [38]; additional work suggests that the parasitophorous vacuole may sometimes fuse directly with the endoplasmic reticulum [14]. Yet another possible model for antigen escape from the parasitophorous vacuole involves vacuolar membrane breakdown by IFN-γ inducible p47 GTPase induced autophagic mediated mechanisms releasing vacuolar proteins into the host cell cytoplasm [15], [39].

The use of antigen-specific reagents, including endogenous T-cell tetramers and T-cell specific mice [12], [16], [17], [40] should provide answers to questions that have been difficult to interpret using model antigen systems, including: the differences in T-cell responses to multiple *T. gondii* strains, the role of changing antigen availability during parasite differentiation, and the generation of memory precursors. It will still be necessary to examine each antigen individually, however, as different antigens can yield different T-cells responses, based not only on their protein sequence but also subcellular location, perhaps influencing the clonality of the immune response. Further studies will be necessary to understand how secreted antigens become accessible to the MHC-I pathway, despite their apparent confinement to the parasitophorous vacuole, and such work is likely to offer new insights into the activation of CD8^+^ T-cells during *T. gondii* infection.

## Materials and Methods

### Ethics statement

Mouse studies were conducted in accord with all relevant national and international guidelines, as approved by the University of Pennsylvania Institutional Animal Care and Use Committee (protocol 801344).

### Parasites and cell cultures

RH ΔUPRT ΔHXPGRT strain *T. gondii* parasites were maintained by serial passage in human foreskin fibroblast (HFF) monolayers, cultivated in Eagle's Minimal Essential Medium (Gibco) containing 1% fetal bovine serum (FBS), as previously described [41]. Extracellular tachyzoites were purified by filtration through 3.0 µm filters (Nuclepore), and washed in phosphate buffered saline.

### Molecular methods

Expression vectors were based on the Bluescript pKS(+)-derived plasmids *tub*P30OVA*dhfr* and *tub*CATOVA*dhfr* described previously by Pepper *et al*, in which the parasite's major surface antigen (P30, encoded by the *SAG1* gene), lacking the C-terminal glycophosphatidyl inositol (GPI) addition signal, is fused to amino acids 140–386 of *Gallus gallus* ovalbumin (OVA) [19]. Transcriptional regulatory sequences were provided by the *T. gondii* tubulin 1 promoter (*tub*) and dihydrofolate reductase 3′ untranslated region (*dhfr*). Plasmids employed in this report substituted the following sequences in place of P30 (between *tub* and OVA, flanked by *Bgl II* and *Avr* II sites): IMC1 (amino acids 1–608) for targeting to the inner membrane complex [42], heat shock protein 60 (HSP 60) for mitochondrial targeting (amino acids 1–55) [43], ROP1 for rhoptry targeting (amino acids 1–396) [44], or MIC3 for microneme targeting (amino acids 1–358) [45]. Retention of P30-OVA in the plasma membrane was achieved by adding the P30 GPI anchor motif (AAGTASHVSIFAMVIGLIGSIAACVA; flanked by *Nhe* I and *Afl* II sites) to the C-terminus of OVA. All plasmids also included a *sag*CAT*sag* cassette downstream of the OVA reporter gene for selecting stable transgenic parasites. 10^7^ freshly harvested extracellular tachyzoites were transfected with 50 µg linearized plasmid DNA (2 mm gap cuvettes, 1.5 kEV pulse, 24Ω), inoculated into HFF cell monolayers in 20 µM chloramphenicol, and incubated through three passages prior to cloning in 96 well plates by limiting dilution [46].

### Immunofluorescence Assays and Microscopy

Confluent HFF monolayers grown on glass coverslips were inoculated with clonal parasite lines expressing OVA, incubated for various times, fixed in 4% formaldehyde (in PBS), permeabilized in 0.2% TritonX-100, blocked in 10% fetal bovine serum, and stained with rabbit anti-chicken ovalbumin (Bethyl Laboratories; Montgomery, Texas), followed by ALEXA 448-conjugated goat anti-rabbit antibodies (Molecular Probes/Invitrogen), as described previously [19]. For co-localization, parasites were stained with mouse anti-P30 or anti-IMC1 (kindly provided by Drs. David Sibley, Washington University School of Medicine, and Gary Ward, University of Vermont, respectively), followed by ALEXA 594-conjugated goat anti-mouse antibodies (Molecular Probes/Invitrogen). Mitotracker was used for mitochondrial detection, and 10 µM DAPI for nuclear labeling. Images were captured using a Leica inverted epifluorescence microscope fitted with a 100 W Hg lamp and appropriate filter sets.

### Western Blots

Free *T. gondii* tachyzoites (2×10^6^) were filtered through a 3 µM Nuclepore filter, pelleted by centrifugation, washed with PBS, and treated 30 min with DNAse I (0.2 U/µl) at 37°C. Parasite samples and OVA standards (Worthington Biochemical; Lakewood NJ), were boiled in reducing agent and loading buffer and separated on a 4–12% bis-tris gel (Nupage; Invitrogen) in MES, in parallel with protein standards (MagicMark; Invitrogen), and transferred to nitrocellulose membranes using a semi-dry system (BioRad). After probing with rabbit anti-OVA (Bethyl Laboratories; 1∶2000) or anti-*Tg*DHFR [47] and horseradish peroxidase-conjugated goat anti-rabbit antibodies (Jackson ImmunoResearch; 1∶3000), chemiluminescence was detected with Immobilon Chemiluminescent HRP Substrate (Millipore; Billerica MA) and exposed to Kodak MR X-ray film. OVA production levels were determined using a standard curve generated from purified OVA dilutions run in parallel with parasite samples, and analyzed using Image J software.

### B3Z assays

Macrophages (MØ) and dendritic cells (DC) were prepared as described previously [48], [49]. Briefly, bone marrow was extracted from C57Bl/6 female mice, and cultivated 8–9 d in uncoated plastic dishes containing RPMI medium, 10% FBS (Gibco), and either 30% L929 culture supernatant (for MØs), or 20 ng/ml GM-CSF (PeproTech, for DCs). Cells were inoculated into 96 well flat bottom plates at 10^5^ per well, incubated overnight at 37°C, supplemented with medium +/− recombinant mouse interferon-γ (100 U/ml), and incubated for 4 hr. Triplicate wells were inoculated with live (MOI = 0.5) or heat-killed (MOI = 5) tachyzoites, recombinant OVA (500 µg/ml; Worthington, Lakewood NJ), or OT-I peptide (50 ng/ml SIINFEKL; CHI Scientific, Maynard MA), and incubated 12 hr in RPMI+10% FBS, followed by the addition of B3Z T-cell hybridoma cells (10^5^ / well), and a 12 hr further incubation in RPMI without phenol red. Finally, cultures were supplemented with 100 µM chlorophenol red ß-D-galactopyranoside (CPRG; Calbiochem), incubated 12 hr, and ß-galactosidase activity assessed by spectrophotometric determination of absorbance at 565 nm. All experiments were conducted in triplicate, averaged, and normalized with reference to controls exposed to neither parasites nor OVA.

### *In vivo* assays

C57Bl/6 and Thy1.1 C57Bl/6 mice were obtained from NCI Production and Jackson Labs, respectively; Thy1.2 OT-I TCR transgenics were bred in an SPF facility at the University of Pennsylvania. Thy 1.2 OT-I T-cells from pooled spleens and lymph nodes of naïve C57Bl/6 female mice were purified on CD3/CD28 columns, labeled 9 min in 5 µM carboxy fluoresceine succinimidyl ester (CFSE; Molecular Probes/Invitrogen) and transferred into congenic Thy 1.1 C57Bl/6 females animals (5×10^5^ cells/mouse). After 24 hr, these animals were infected with 10^4^ *T. gondii* parasites (RH strain or RH-OVA transgenics) with 3 mice per group. CFSE levels were determined by flow cytometry (FACs Canto, Becton-Dickinson) at d3, 4 and 5 post-infection, in order to assess the proliferation of OT-I cells. CD8^+^ T-cell activation was determined in parallel using antibodies against CD62L (eBiosciences clone Mel-14), CD44 (eBioscience clone IM7), and CD69 (eBiosciences clone H1.2F3). Data collection and analysis was carried out using DIVA and FlowJo software. *In vivo* parasite burden was tested using real-time PCR as described previously [50]. Briefly, parasite genomic DNA was isolated using the High Pure PCR Purification Kit (Roche) and real-time analysis conducted using B1 primers on a 7500 Fast Real-Time PCR System.

### Statistical analysis

Student's T tests were completed for the B3Z and flow cytometry assays using GraphPad Prism software. P values<0.05 (*) or <0.005 (**) are indicated.
